# A unified design approach for control integrating processes with time delay

**DOI:** 10.1371/journal.pone.0299893

**Published:** 2024-06-13

**Authors:** Chengqiang Yin, Shourui Wang, Jie Gao

**Affiliations:** 1 School of Machinery and Automation, Weifang University, Weifang, Shandong Province, China; 2 School of Mechanical and Electrical Engineering, Lanzhou University of Technology, Lanzhou, Gansu Province, China; National Institute of Technology Silchar, India, INDIA

## Abstract

The article presents a unified control system designing scheme to obtain enhanced performance for processes including integrator and dead time. A simple control structure including two controllers is proposed. Servo performance and disturbance rejection performance can be adjusted independently by introducing a desired transfer function model in the control structure. Servo controller is designed according to the direct synthesis principle and disturbance rejection controller is derived adopting the internal model control (IMC) theory. Simulations have been conducted on four kinds of integrating plants with dead time. The simulation results exhibit that noteworthy enhancement can be achieved by the presented scheme in comparation with the other methods even though there are perturbed dynamics.

## Introduction

In process industry, integrating processes are often encountered and regarded one of the relatively challenging difficulties to control especially for the presence of time delays. In some cases, an excessive overshoot and long settling time will occur because of the existence of integrating factor, more seriously, balance of the system is easily damaged [1, 2].

Numerous control schemes were presented for processes including integrator and dead time in research literatures. Proportional Integral Derivative (PID) control is one of the effective methods employed mostly in many fields because of its simplicity, but disturbance rejection and robustness have not fully achieved by conventional PID tuning methods in single closed loop structure. So some effective tuning methods have been proposed, IMC-PID based tuning algorithms are widely presented for integrating systems [3–5]. Such as Praveen [6] designed a control scheme with PID controller and filter using sensitivity of transfer function for various classes of integrating process. In literature [7], a controller with set point weighting was designed using direct synthesis method. Zhang et al. [8] proposed an optimal and analytical design procedure based on the empirical method with modified Smith predictor (MSP) structure [9]. They tuned the parameter according to the desired frequency domain properties or time domain criteria. According to IMC *H*_2_ minimization theory Ghousiya Begum et al [10] designed a control scheme for processes with integrator and dead time. IMC-PID based tuning algorithms exhibit their superiority, however the standard IMC-PID controller provides only good servo performance but poor disturbance rejection response.

For the single loop control structure, water-bed phenomenon is inevitable in the performances of servo and disturbance restraining. To overcome the deficiency, much two degree of freedom control methods were proposed. Wang et al. [11] presented a control scheme in discrete time form. The servo controller was designed according to the optimal control method based on *H*_2_ principle, the other controller was developed through designing the transfer function. What’s more, methods on the basis of Smith predictor structures have been presented and demonstrated good performance for disturbance rejection as well as servo property [12–15]. Such as the control scheme for processes including integrator was proposed in [16], the two controllers were all designed as PD form utilizing the rules of gain and phase margin. Ajmeri [17] presented a novel structure to control the integrating process with dead time. In their work, servo controller was designed as PD form and disturbance rejection controller was designed as PID form, the two controllers were all obtained using the desired transfer function. To deal with the influence of inverse response for integrating process with dead time, Proportional Integral (PI)-Proportional Derivative (PD) design scheme incorporating the Smith predictor structure was proposed in literature [18]. For the first integrating and dead time processes, Somak et al. [19] presented a control scheme with two controllers, and set point weighing was introduced in the set point tracking controller. After that, they modified the previous control scheme for second order integrating processes [20], and the parameters for the two controllers were designed according to the IMC tuning guideline and Routh stability analysis respectively. Superiority of the control scheme was demonstrated by comparison with others’ methods. For double integrating with time delay processes, Sengupta et al. presented a control scheme based on modified smith predictor. Two fractional PD controllers were designed for servo response and load regulation, and a first-order filter was designed for providing improved robustness [21].

Cascade control is an alternative method can enhance the system capability obviously. Lloyds Raja [22] proposed a method on the basis of the MSP taking advantage of the merit of the structure of the cascade control. Routh–Hurwitz stability theory and IMC were adopted to derive the three controllers. Similarly, in the control scheme [23], loop decomposition was used to split the outer loop model into two models, PI controller was designed using moment matching method and proportional controller was used for the integrating element. In literature [24], a unified control scheme based on classical cascade control structure was proposed for integrating process. By setting suitable poles, the enhanced disturbance rejection performance could be obtained.

What’s more, some advanced control principle have been introduced to deal with the integrating process with time delay. Such as Mehta et al. presented a control scheme including fractional-order integral derivative controllers, complex root boundary analysis and three-step optimization algorithm were used to set tuning parameters [25]. Kaya and Cokmez proposed the design method of integral–proportional derivative controller for integrating processes. The controllers were obtained using analytical rules based on curve fitting techniques [26]. Huba et al. extended the reference model-based dead-time compensator for double integrator with time delay system, the control performance is excellent although the method is complicated [27].

It can be seen from the existing research results that the two degree of freedom control had exhibited excellent performance compared with the unity feedback control. But as for the most control schemes, the controllers were derived using different methods, which added the complexity of design. In this study, a unified approach is presented for first and second order processes including integrator and dead time. A control scheme depending on a simple control structure and a unified deriving procedure is proposed for different integrating processes. The servo controller is derived through introducing the simple and effective transfer function. And IMC-PID design principle is adopted to derive the disturbance rejection controller, which reduces complexity of controller design as well as improves the system performance. As a result, complete decoupling is achieved between the responses of set point tracking and load disturbance rejection, and the two responses can be tuned individually through two parameters. Compared to the control methods presented recently, the control scheme proposed in this study demonstrates enhanced capabilities observably. Organization of this paper is: control structure and plant models are introduced in Proposed control scheme section. Controllers designing methods are detailed in Controller design section. Suggestions for setting the controller parameters are presented in Guidelines for adjustable parameters section. And robust stability analysis of the control system is in the following section. Effectiveness of the suggested control scheme is exhibited in Simulation studies section by introducing four different examples. Some conclusions are drawn in the last section.

## Proposed control scheme

The classes of the processes including integrator and dead time studied in the present work are

Case 1: Integrator plus time delay (IPTD)
$$P(s)=\frac{ke^{-\theta s}}{s}$$ (1)
Case 2: Double integrator plus time delay (DIPTD)
$$P(s)=\frac{ke^{-\theta s}}{s^{2}}$$ (2)
Case 3: First order stable with integrator and time delay (FOSITD)
$$P(s)=\frac{ke^{-\theta s}}{s(Ts+1)}$$ (3)
Case 4: First order unstable with integrator and time delay (FOUITD)
$$P(s)=\frac{ke^{-\theta s}}{s(Ts-1)}$$ (4)

**Fig 1:**
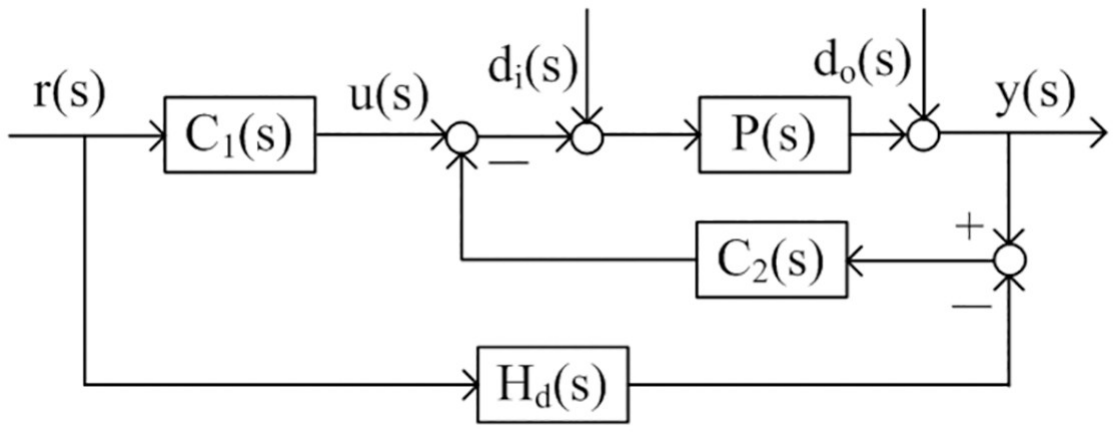
The proposed structure of the control scheme.

For the plants with model given above, a simple control structure is presented as Fig 1. The principle of the control scheme is to simplify the design procedure and realize the set point tracking performance and disturbance rejection performance of the system can be optimized separately. To improve the set point tracking performance, a specific transfer function between the input and output is designed in the structure. The detailed description of the control structure is given as follows. *P*(*s*) denotes the actual process, *r*(*s*) denotes the reference input, *y*(*s*) is the process output, *d*_*i*_(*s*) denotes the disturbance before the process, *d*_*o*_(*s*) is the disturbance after the process. *H*_*d*_(*s*) is the designed function describing the relation of output *y*(*s*) with the reference input *r*(*s*). *C*_1_(*s*) denotes the servo controller, it plays a part in improving the set point tracking performance, *C*_2_(*s*) denotes the disturbance rejection controller, it is responsible for stability and load disturbance rejection. According to the law of signal transmission in Fig 1, the following two transfer functions can be got as

$$\frac{y(s)}{r(s)}=\frac{\left(C_{1}(s)+C_{2}(s)H_{d}(s))P(s\right)}{C_{2}(s)P(s)\text{+}1}$$ (5)

$$\frac{y(s)}{d_{i}(s)}=\frac{P(s)}{1+C_{2}(s)P(s)}$$ (6)

The transfer function expressed by Eq (5) describes the relation between the input and the output of the system. And the transfer function deduced by Eq (6) describes the relation between the disturbance and the output of the system. Based on the two transfer functions, the servo controller and the disturbance rejection controller will be obtained by theoretical analysis. At the same time, it can be seen from Eqs (5) and (6) that the performance of disturbance rejection is related to the controller *C*_2_(*s*), but the servo response is decided by the two controllers. However, we will observe that only controller *C*_1_(*s*) takes care of servo response if suitable transfer function *H*_*d*_(*s*) is introduced.

## Controller design

### Servo controller design

In order to decouple completely the servo response and disturbance rejection response, and simplify the procedure of the controller design, we suppose *y*(*s*)/*r*(*s*) = *H*_*d*_(*s*), which will remove the effect of the controller *C*_2_(*s*) on the servo performance. The objective of design *C*_1_(*s*) is to provide a stable set point tracking performance with strong robustness. The desired function describing the relation of output *y*(*s*) with reference input *r*(*s*) will be specified for the controlled process, and the servo controller can be obtained according to the direct synthesis method.

Direct synthesis is a method used to design controller, in general, it relies on both the model of the process and the desired system response. Most of all, the controller is designed according the desired output behavior of the closed loop system which is specified as a trajectory model based on the process. And the controller parameters are determined by analytical processing using the desired closed-loop response [28]. With this method, the servo controller will be developed for four types of integrating plant. Firstly, the desired transfer function is designed for the closed loop system as

$$H_{d}(s)=\frac{1}{{\left(\lambda_{1}s+1\right)}^{n}}e^{-\theta s}$$ (7)

As can be seen from the Eq (7) that the desired transfer function contains an adjustable parameter *λ*_1_ and a dead time term *e*^−*θs*^. The parameter *λ*_1_ is used to adjust the set point tracking velocity of the system. The dead time term is the same as the delay of the controlled process and can’t be eliminated because of its essential characteristic. By designing the above form of *H*_*d*_(*s*), we can see that the servo response is stable and the design method is executable. What’s more, the servo response will be accurate extremely if *λ*_1_ is tuned to zero. In the desired transfer function, *n* is related to the order of the plant, for the process with one integrator and dead time, *n* = 2 is designed. For the other three integrating processes described in Eqs (2)–(4), *n* = 3 is designed. So the servo controller *C*_1_(*s*) can be got on the basis of the assumption *P*(*s*) *C*_1_(*s*) = *H*_*d*_(*s*).


$$C_{1}(s)=\frac{1}{{\left(1\text{+}\lambda_{1}s\right)}^{n}P(s)}e^{-\theta s}$$ (8)

From the design procedure for controller *C*_1_(*s*) we can see that the servo response is only concerned with *C*_1_(*s*) and the servo response and disturbance rejection response can be adjusted by controllers *C*_1_(*s*) and *C*_2_(*s*) respectively.

### Disturbance rejection controller design

As we know, for the control process with integrator and dead time, output of the system will vary rapidly or oscillate dramatically when it is affected by a big external disturbance. In this work, to improve the disturbance rejection performance, integrator is converted into unstable block while designing the disturbance rejection controller. Such as *ke*^−*θs*^/*s* can be converted as *kTe*^−*θs*^/(*Ts* − 1), T is a bigger constant. And analytical method based on the IMC principle is adopted for deriving the disturbance rejection controller.

**Fig 2:**
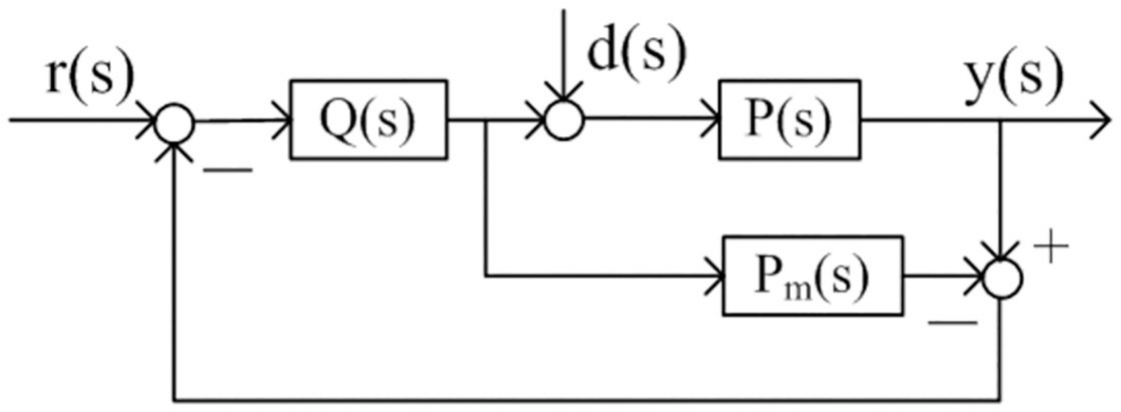
IMC control structure.

**Fig 3:**
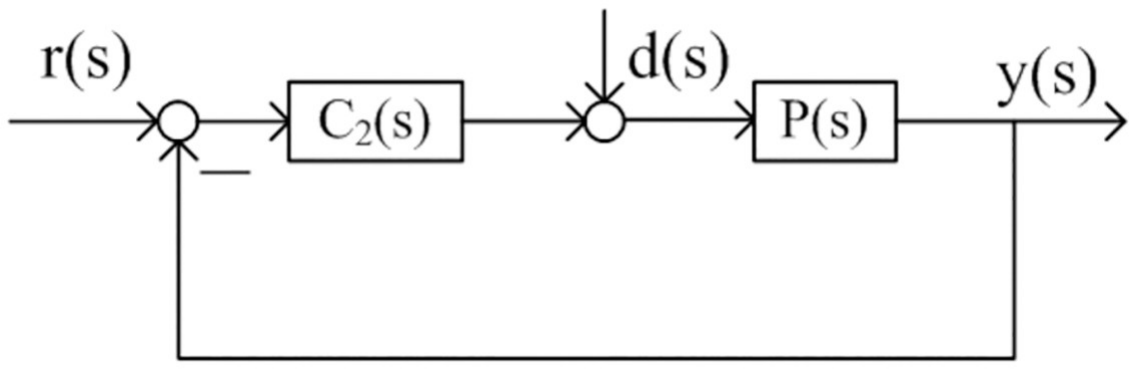
Feedback control structure.

Tuning methods using IMC-PID theory are simple and convenient [29, 30]. Figs 2 and 3 show the IMC diagram and single loop control diagram respectively. In these two figures *P*(*s*) denotes the control process, *P*_*m*_(*s*) denotes the model of the process, *Q*(*s*) denotes the internal model controller, *C*_2_(*s*) denotes the feedback controller based on the controller *Q*(*s*). The transfer function in the nominal case (*P*(*s*) = *P*_*m*_(*s*)) for the Fig 2 is

$$\frac{y(s)}{r(s)}=P(s)Q(s)$$ (9)

The idea of IMC is to connect the process model in parallel with the process, and the internal model controller is obtained by approximating the dynamic inverse of the model. For single input and single output system, the controller is designed using the inverse of the minimum phase part of the model and adding a low-pass filter to enhance the robustness of the system. Adopting the design procedure of IMC, the model of the process *P*_*m*_(*s*) can be expressed as *P*_*m*_(*s*) = *P*_*m+*_(*s*) *P*_*m*−_(*s*), *P*_*m+*_(*s*) denotes the noninvertible section and *P*_*m*−_(*s*) denotes the invertible section of the model. According the IMC design method, the controller *Q*(*s*) is designed as $Q(s)=P_{m-}^{-}(s)f_{\text{IMC}}(s)$, where *f*_*IMC*_(*s*) is a filter, it is selected to make *Q*(*s*) stable, proper and realizable.

$$f_{IMC}(s)=\frac{\sum_{i=1}^{n}b_{i}s^{i}+1}{{\left(\lambda_{2}s+1\right)}^{r}}$$ (10)

Where *n* is the amount of unstable poles, a big *r* is needed to get a realizable internal model controller, *λ*_2_ is the adjustable parameter. *b*_*i*_ is obtained according to $1-P(s)Q(s){|}_{s=p_{1}\cdots p_{n}}=0$, where *p*_1_ … *p*_*n*_ are the unstable poles. So the IMC controller can be got as

$$Q(s)=\frac{P_{m-}^{-1}(s)(\sum_{i=1}^{n}b_{i}s^{i}+1)}{{\left(\lambda_{2}s+1\right)}^{r}}$$ (11)

At the same time, according to the design scheme shown in Fig 1, the complementary sensitivity function of the system is derived as

$$T(s)=\frac{P(s)C_{2}(s)}{P(s)C_{2}(s)\text{+}1}$$ (12)

We can observe that the complementary sensitivity function in Fig 1 is equal to the transfer function from the system output *y*(*s*) to the reference input *r*(*s*) in Fig 3. Therefore, the controller *C*_2_(*s*) can be obtained in the light of the idea of IMC theory based single loop control, that is

$$\frac{P(s)C_{2}(s)}{P(s)C_{2}(s)\text{+1}}=P(s)Q(s)$$ (13)

On substituting Eq (11) into Eq (13), the disturbance rejection controller *C*_2_(*s*) can be obtained

$$C_{2}(s)=\frac{P_{m-}^{-1}(s)(\sum_{i=1}^{m}b_{i}s^{i}+1)}{{\left(\lambda_{2}s+1\right)}^{r}-P_{m+}(s)(\sum_{i=1}^{m}b_{i}s^{i}+1)}$$ (14)

For the process with an integrator and dead time, we transform it as *Tke*^−*θs*^/(*Ts* − 1) and set *r* = 3. The simplified expression for the controller *C*_2_(*s*) is

$$C_{2}(s)=\frac{\left(Ts-1)(bs+1\right)}{Tk[{\left(\lambda_{2}s+1\right)}^{3}-e^{-\theta s}(bs+1)]}$$ (15)

Where *b* = *T*[(1 + *λ*_2_/*T*)^3^*e*^*θ*/*T*^ − 1]. To overcome consequence brought about by the dead time term in denominator and make the controller realizable, dead time term *e*^−*θs*^ is approximated using Pade expansion

$$e^{-\theta s}=\frac{6-2\theta s}{\theta^{2}s^{2}+4\theta s+6}$$ (16)

Substituting Eq (16) into Eq (15) obtains the disturbance rejection controller as

$$C_{2}(s)=\frac{\left(Ts-1)(bs+1)(\theta^{2}s^{2}+4\theta s+6\right)}{Tk[{\left(\lambda_{2}s+1\right)}^{3}(\theta^{2}s^{2}+4\theta s+6)-(bs+1)(6-2\theta s)]}$$ (17)

To convert the controller *C*_2_(*s*) into a PID form and avoid losing of accuracy, we rearranged Eq (17) as

$$C_{2}(s)=\frac{\theta^{2}s^{2}+4\theta s+6}{Tk\eta s}\times \frac{\left(bs+1)(Ts-1\right)}{l_{4}s^{4}+l_{3}s^{3}+l_{2}s^{2}+l_{1}s+1}$$ (18)

where *η* = 18*λ*_2_ + 6*θ* − 6*b*, $l_{4}=\lambda_{2}^{3}\theta^{2}/\eta$, $l_{3}=(4\lambda_{2}^{3}\theta +3\lambda_{2}^{2}\theta^{2})/\eta$, $l_{2}=(6\lambda_{2}^{3}+12\lambda_{2}^{2}\theta +3\lambda_{2}\theta^{2})/\eta$,$l_{1}=(18\lambda_{2}^{2}+12\lambda_{2}\theta +\theta^{2}+2b\theta )/\eta$.

The controller shown in Eq (18) is expressed in PID form with a filter [31].

$$C_{2}(s)=K_{p}(1+\frac{1}{T_{i}s}+T_{\text{d}}s)\frac{1+\zeta s}{1+\psi s}$$ (19)

Where *K*_*p*_ = −4*θ*/*Tkη*, *T*_*i*_ = 2*θ*/3, *T*_*d*_ = *θ*/4, *ζ* = *b*, *ψ* = *l*_1_ + *T*.

According to the design method proposed above, the other three integrating processes described in Eqs (2)–(4) are represented as a general process model

$$P(s)=\frac{K}{\left(T_{1}s-1)(T_{2}s-1\right)}e^{-\theta s}$$ (20)

After transformation, we can obtain the process gain *K* and the time constants. *T*_1_, *T*_2_ can be got by converting integrator to unstable block. Subsequently, we will present the design procedure of the disturbance rejection controller for the process as Eq (20).

The controller *C*_2_(*s*) can be obtained using Eq (14) by setting *r* = 4

$$C_{2}(s)=\frac{\left(1+c_{1}s+c_{2}s^{2})(T_{1}s-1)(T_{2}s-1\right)}{K[{\left(\lambda_{2}s+1\right)}^{4}-e^{-\theta s}(1+c_{1}s+c_{2}s^{2})]}$$ (21)

Where *c*_1_ and *c*_2_ can be obtained by the following two constraints

$$\underset{s\to 1/T_{1}}{\text{lim}}[1-\frac{c_{2}s^{2}+c_{1}s+1}{{\left(1\text{+}\lambda_{2}s\right)}^{4}}e^{-\theta s}]=0,\;\;\;\;\underset{s\to 1/T_{2}}{\text{lim}}[1-\frac{c_{2}s^{2}+c_{1}s+1}{{\left(1+\lambda_{2}s\right)}^{4}}e^{-\theta s}]=0$$ (22)

Following a simple calculation, *c*_1_ and *c*_2_ can be obtained as

$$c_{1}=\frac{T_{2}^{2}{\left(\frac{\lambda_{2}}{T_{2}}+1\right)}^{4}e^{\theta /T_{2}}-T_{1}^{2}{\left(\frac{\lambda_{2}}{T_{1}}+1\right)}^{4}e^{\theta /T_{1}}+T_{1}^{2}-T_{2}^{2}}{\left(T_{2}-T_{1}\right)},\;\;\;\;c_{2}=T_{2}^{2}\left[{\left(\frac{\lambda_{2}}{T_{2}}+1\right)}^{4}e^{\theta /T_{2}}-1\right]-c_{1}T_{2}$$ (23)

To overcome consequence brought about by the dead time in Eq (21), Pade expansion is used as *e*^−*θs*^ = (1 − *θs*/2)/(1 + *θs*/2). So the controller *C*_2_(*s*) can be obtained substituting Eq (23) into Eq (21) as

$$C_{2}(s)=\frac{c_{2}s^{2}+c_{1}s+1}{\eta }\times \frac{\left(T_{1}s-1)(T_{2}s-1)(1+\theta s/2\right)}{l_{4}s^{4}+l_{3}s^{3}+l_{2}s^{2}+l_{1}s+1}$$ (24)

Where *η* = 4*λ*_2_ − *c*_1_ + *θ*, $l_{1}=(6\lambda_{2}^{2}+2\lambda_{2}\theta +c_{1}\theta /2-c_{2})/\eta$, $l_{2}=(4\lambda_{2}^{3}+3\lambda_{2}^{2}\theta +c_{2}\theta^{2}/2)/\eta$,$l_{3}=(\lambda_{2}^{4}+2\lambda_{2}^{3}\theta )/\eta$, $l_{4}=\lambda_{2}^{4}\theta^{2}/2\eta$. Similarly, the form same with Eq (19) is designed and the parameters are derived as follow

$$K_{p}=c_{1}/k\eta ,\;T_{i}=c_{1},\;T_{d}=c_{2}/c_{1},\;\zeta =0.5\theta ,\;\psi =l_{1}+T_{1}+T_{2}$$ (25)

## Guidelines for adjustable parameters

Selecting a proper controller parameter is important to obtain a better system performance. For the servo controller, we can see from Eq (7) that the tuning parameter acts directly on the servo response. When *λ*_1_ is small, the speed of servo response is fast, and vice visa. In the meantime, the servo response speed is related to the necessary energy from the servo controller. The faster response needs more energy from the controller, which will lead to a more aggressive action when there is an actual plant uncertainty. Generally, to obtain the best compromise between the servo response speed and out capacity of the controller, *λ*_1_ is recommended to adjust around the dead time value of the plant at start. With regards to the disturbance rejection controller, decreasing *λ*_2_ will improve the disturbance rejection performance but will degrade robust stability when there is process uncertainty, and vice visa. So *λ*_2_ can be selected following a principle that the nominal performance of the system keeps balance with robust stability. After simulations for various processes, it is concluded that the value of *ψ* obtained according to the Eqs (19) or (25) is large relatively. To get enhanced capability of disturbance rejection and robust stability, the value of *ψ* can be selected 0.1–0.5 times the obtained value according to Eqs (19) or (25) during application.

## Robust stability analysis

The two controllers *C*_1_(*s*), *C*_2_(*s*) are derived according to the known plant model. In practical application, there are inevitably differences or uncertainties between the used model and the practical system. Therefore, it is essential to conduct robust stability analysis. According to the models used for the controllers, the parameter uncertainties mainly include uncertainty in dead time, time constant, and process gain.

In the light of the small gain theorem, the necessary and sufficient condition for robust stability of a control system is ${\left\|\Delta_{T}(s)T(s)\right\|}_{\infty }<1$, where *T*(*s*) denotes the complementary sensitivity function of the system, Δ_*T*_(*s*) is the bound of the process uncertainty, Δ_*T*_(*s*) = (*P*(*s*) − *P*_*m*_(*s*))/*P*_*m*_(*s*).

If there is uncertainty in the gain of the model, the condition of the robust stability should be satisfied as

$${\left\|T(s)\right\|}_{\infty }<\left|\frac{1}{\left|\Delta k\right|/k}\right|$$ (26)

If there is uncertainty in the dead time of the model, the condition of the robust stability should be satisfied as

$${\left\|T(s)\right\|}_{\infty }<\left|\frac{1}{e^{-\Delta \theta s}-1}\right|$$ (27)

If there are uncertainties both in the gain and in the dead time, the robust stability condition should be satisfied as

$${\left\|T(s)\right\|}_{\infty }<\left|\frac{1}{(\Delta k/k+1)e^{-\Delta \theta s}-1}\right|$$ (28)

For a control system, it is expected to get excellent stability as well as good nominal performance, the constraint principle should be meet as [30]

$$\left|T(s)\Delta_{T}(s)\right|+\left|S(s)W(s)\right|<1$$ (29)

where *S*(*s*) is the closed loop sensitivity function *S*(*s*) = 1 − *T*(*s*), *W*(*s*) is the weight function of *S*(*s*). For the load disturbance characterized with step change, the weight function is set as 1/s in general. In addition, complementary sensitivity is an effective method to demonstrate the insensitive property of a controller when there are parametric uncertainties between practical process and used plant model. In the following examples, complementary sensitivity function will be adopted to show the robust stability of the proposed control scheme.

## Simulation studies

In this section, processes with integrator plus dead time are borrowed from the recently published literatures to verify the superiority performance obtained by the proposed control scheme. Simulations are executed in SIMULINK environment. For quantitative comparison, several performance indices are employed to evaluate the capability of the control scheme including output of the system and output of the controller, such as integral of absolute error (IAE), $IAE=\int_{0}^{\infty }\left|e(t)\right|dt$, integral square error (ISE), $ISE=\int_{0}^{\infty }e^{2}dt$, integral time-weighted absolute error (ITAE), $ITAE=\int_{0}^{\infty }t\left|e(t)\right|dt$, and total variation (TV),$TV=\sum_{n=0}^{N}\left|u_{n+1}-u_{n}\right|$. For the four performance indices, smaller value denotes the superior performance of the control method.

### Exa.1 Integrator plus time delay

**Fig 4:**
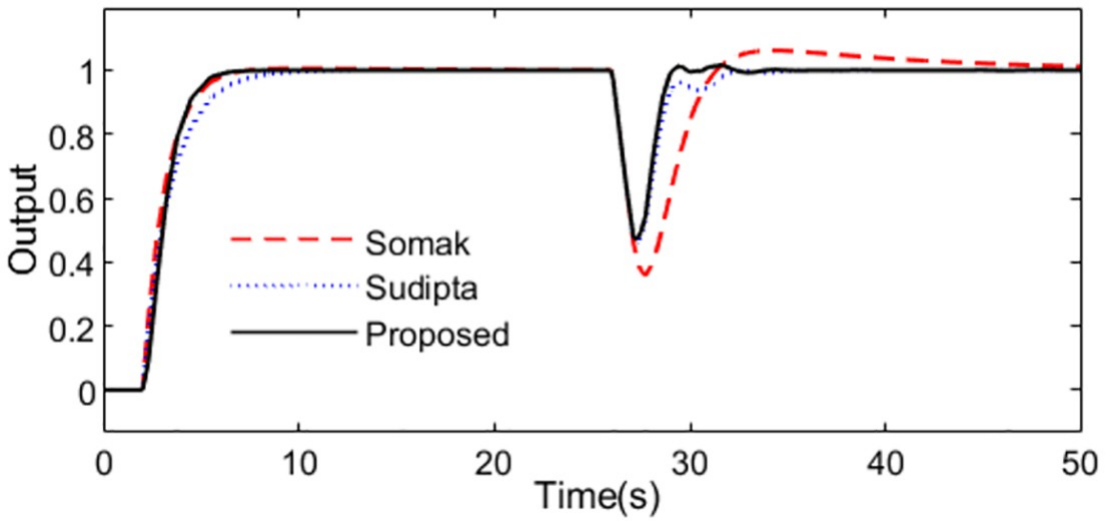
System output in normal case of Exa.1.

**Fig 5:**
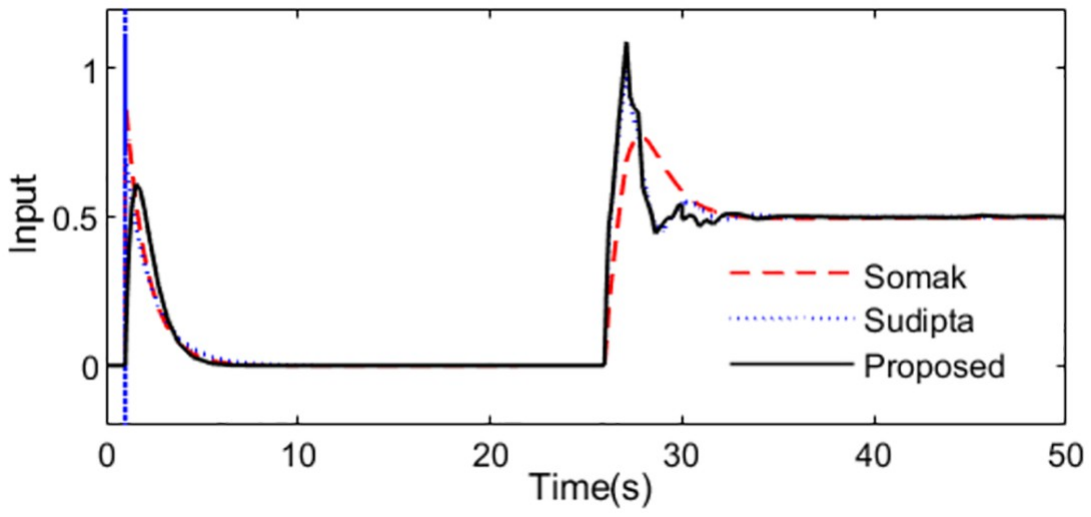
Control action in normal case of Exa.1.

The model for the studied control process is *P*_*m*_(*s*) = *e*^−*s*^/*s*. According to Eq (8), the servo controller is got *C*_1_(*s*) = *s*/(0.6*s* + 1)^2^ by setting the tunning parameter *λ*_1_ = 0.6. To obtain the disturbance rejection controller using Eq (19), the process model is approximated as. *P*_*m*_(*s*) = 100*e*^−*s*^/(100*s* − 1). For comparison, methods proposed by Somak Karan [19] and Sudipta [16] are considered. Somak Karan proposed a modified Smith control structure including two controllers, and set point weighting was introduced in the feed forward path. *G*_*cm*1_ = 1 + 10/*s*, *G*_*cm*2_ = 0.52 + 0.13*s*. Sudipta designed two PD controllers based on MSP control structure *G*_*c*_ = *G*_*d*_ = 0.19(1 + 0.34*s*). With these controller settings, simulations are performed by setting *r*(*s*) as a unit step signal at time t = 1s and setting *d*_*i*_(*s*) as a step signal with magnitude -0.5 at time t = 25s. The system outputs and the inputs to the process are exhibited as Figs 4 and 5 in the normal case.

From the Fig 4, we can see that the output from the presented control system is slightly accurate and steady than that from the other two methods. Fig 5 is the control actions from the controllers. We can see that the output of the controller from the control scheme [19] is steady, which also verified in Tab.1, though other evaluating indexes are excellent for the proposed method.

**Fig 6:**
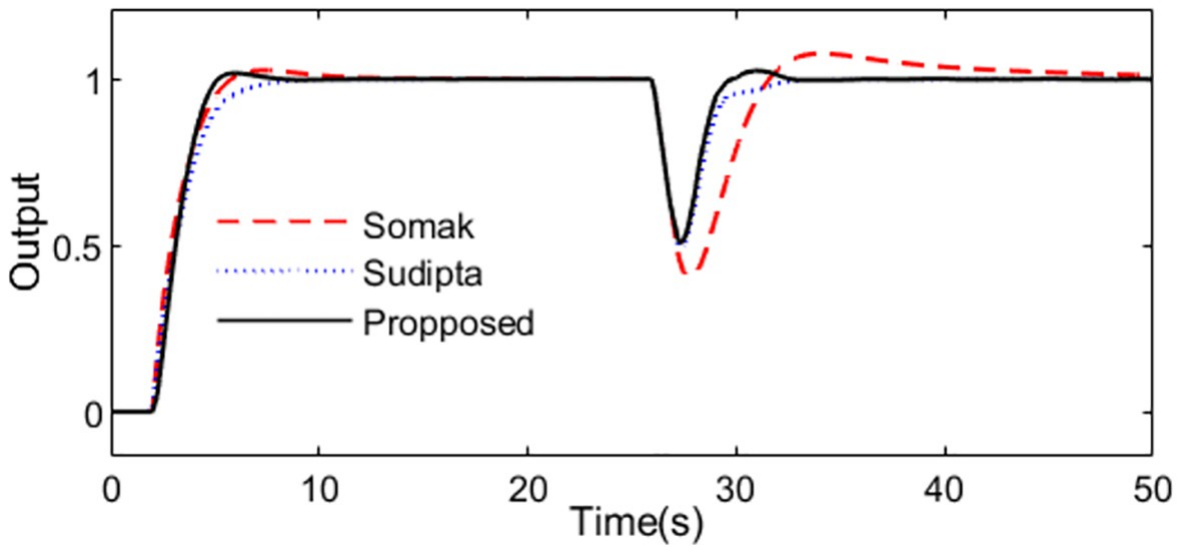
System output in perturbed case of Exa.1.

**Fig 7:**
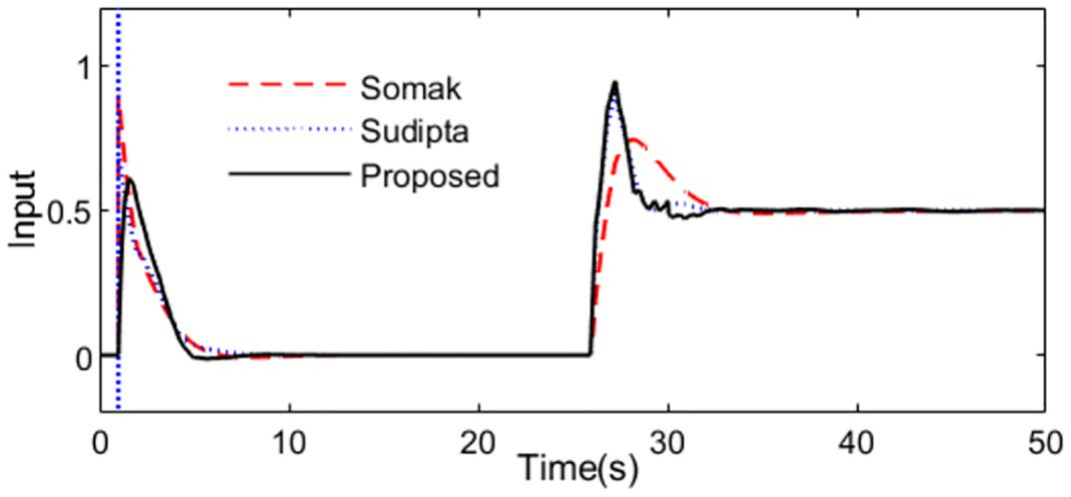
Control action in perturbed case of Exa.1.

What’s more, 10% increment for time constant is supposed to analyze the robustness to the model uncertainties, the system outputs are demonstrated as Fig 6. We can see that the presented control scheme is relatively insensitive to the uncertainty of the parameter comparing with the control schemes presented by Somak Karan and Sudipta. The outputs of the controllers are shown in Fig 7.

**Fig 8:**
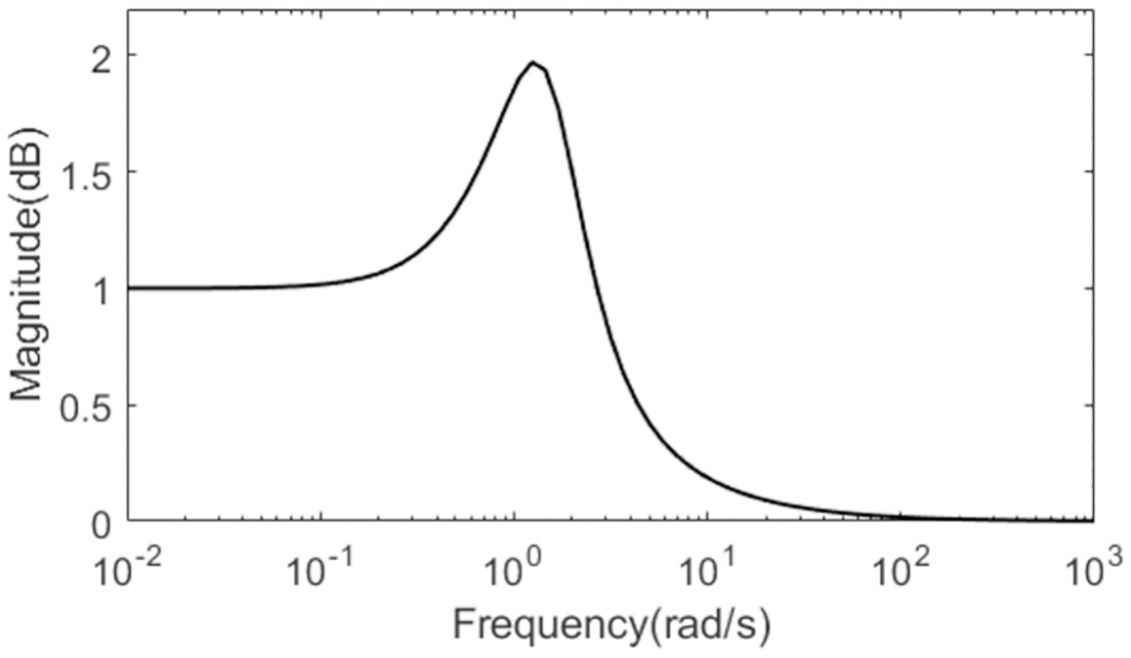
Magnitude of *T*(*s*) of Exa.1.

For quantitative comparison, all the evaluation indexes are obtained in Table 1 for all the methods in normal and perturbed cases. Obviously, the evaluation indexes calculated for the presented scheme are smaller besides the TV. To represent clearly the characteristic of the complement sensitivity function, the magnitude of *T*(*s*) for the presented control scheme is illustrated in Fig 8.

**Table 1:** Evaluation indexes of Exa.1.

State	Method	IAE	ISE	ITAE	TV
Normal	Somak	4.50	2.37	78.62	2.17
	Sudipta	3.49	2.06	35.91	106
	Proposed	3.14	2.06	30.83	5.80
Perturbed	Somak	4.72	2.43	82.48	2.17
	Sudipta	3.46	2.10	35.51	108
	Proposed	3.20	2.10	31.20	5.02

### Exa.2 Double integrator plus time delay

The difficult and challenging process controlled by Somak [20] is studied here *P*_*m*_(*s*) = *e*^−5*s*^/*s*^2^. In literature [20], they proposed a modified Smith predictor structure including two PD controllers and a filter as *G*_*c*1_ = 0.64(1 + 2.5*s*), *G*_*c*2_ = 0.007(1 + 19.53*s*), *F* = 1/(1 + 0.0125*s*). By using Eq (8), the servo controller is got as *C*_1_(*s*) = *s*^2^/(0.8*s* + 1)^3^ by setting the tunning parameter *λ*_1_ = 0.8. To obtain the disturbance restraining controller using Eq (25), the process model is approximated as *P*_*m*_(*s*) = −10000*e*^−5*s*^/(100*s* − 1)(−100*s* − 1). And the controller is designed as follow by selecting *λ*_2_ = 3.


$$C_{2}(s)=0.0311(1+\frac{0.0586}{s}+7.4264s)\frac{1+2.5s}{1+0.1818s}$$

**Fig 9:**
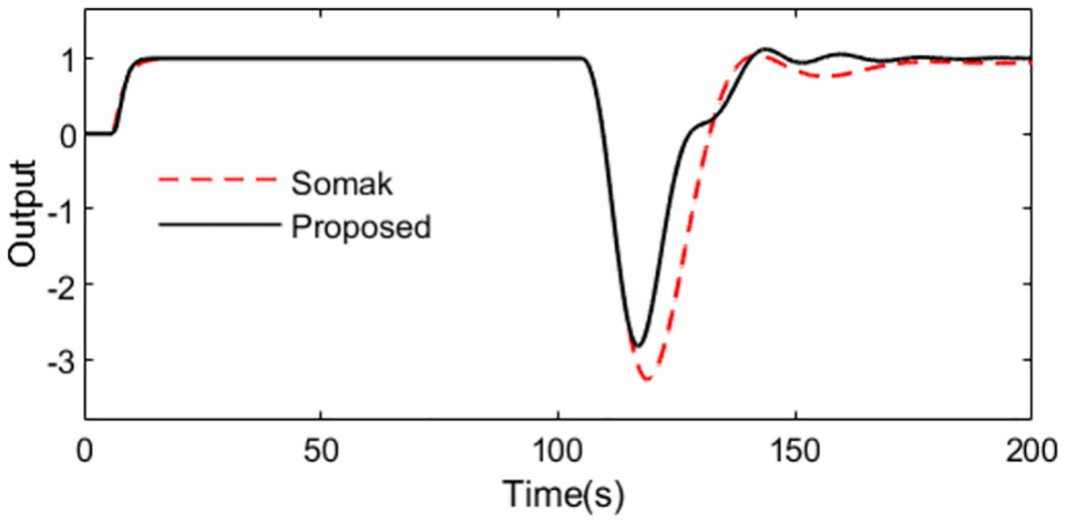
System output in normal case of Exa.2.

**Fig 10:**
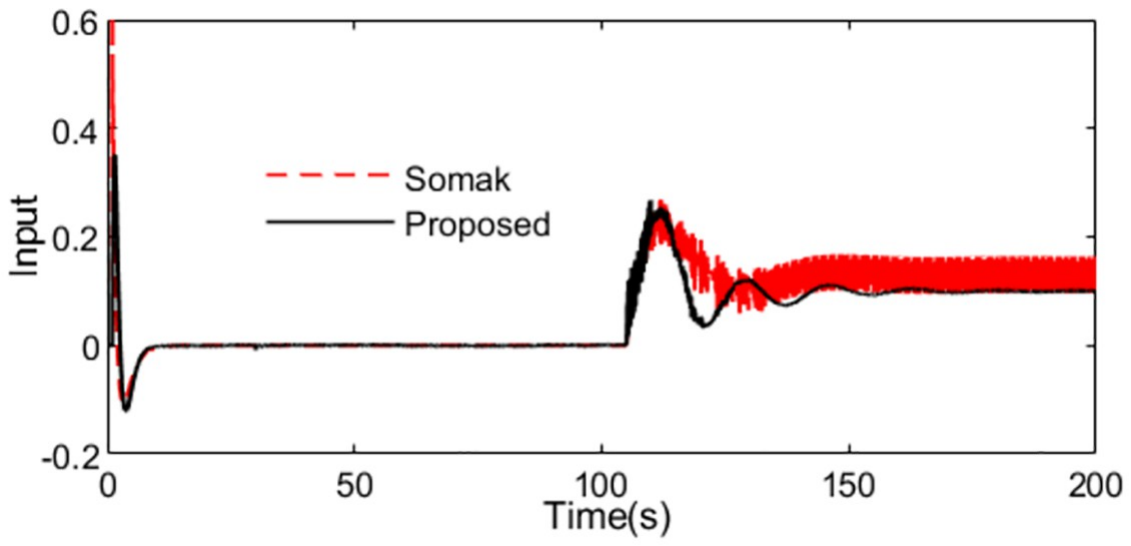
Control action in normal case of Exa.2.

With these controller settings, simulations are performed by setting *r*(*s*) as a unit step signal at time t = 1s and setting *d*_*i*_(*s*) as a step signal with magnitude -0.1 at time t = 100s. The system outputs and the inputs to the process are shown as Figs 9 and 10 in the normal case. The figures show that the set point tracking capability of the proposed scheme is more perfect than Somak’s method, and the control action for the presented control scheme is obviously smoother than that of Somak’s method.

**Fig 11:**
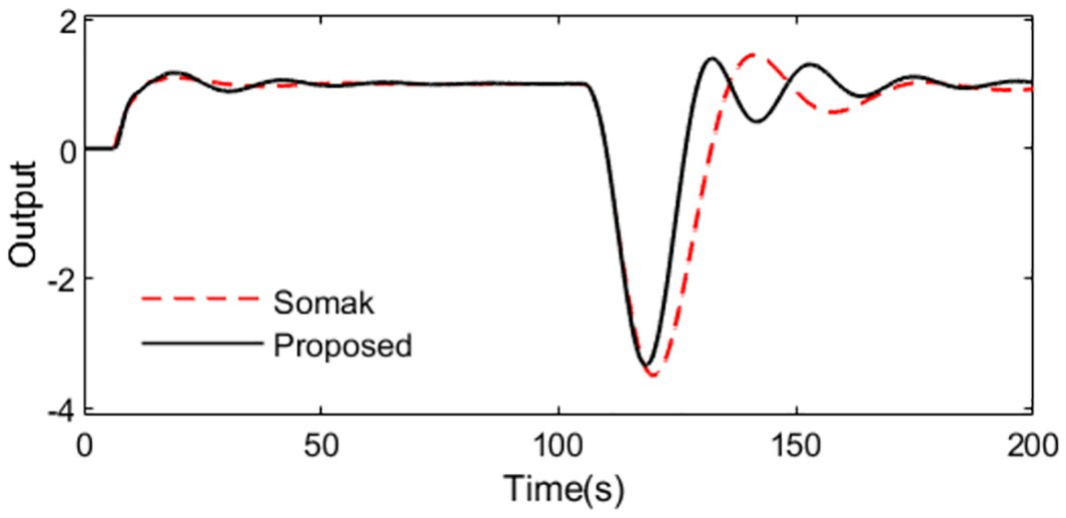
System output in perturbed case of Exa.2.

**Fig 12:**
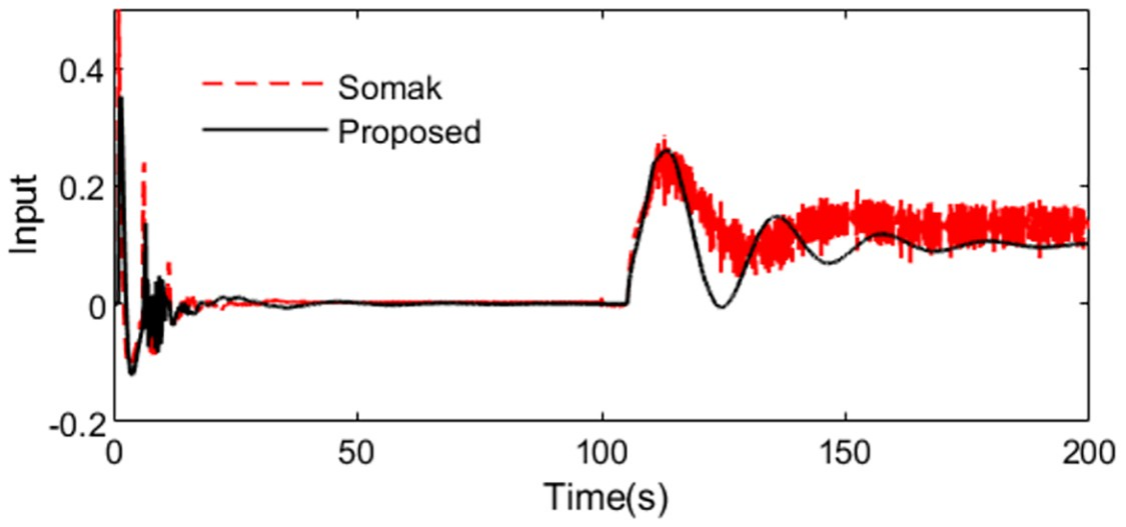
Control action in perturbed case of Exa.2.

**Fig 13:**
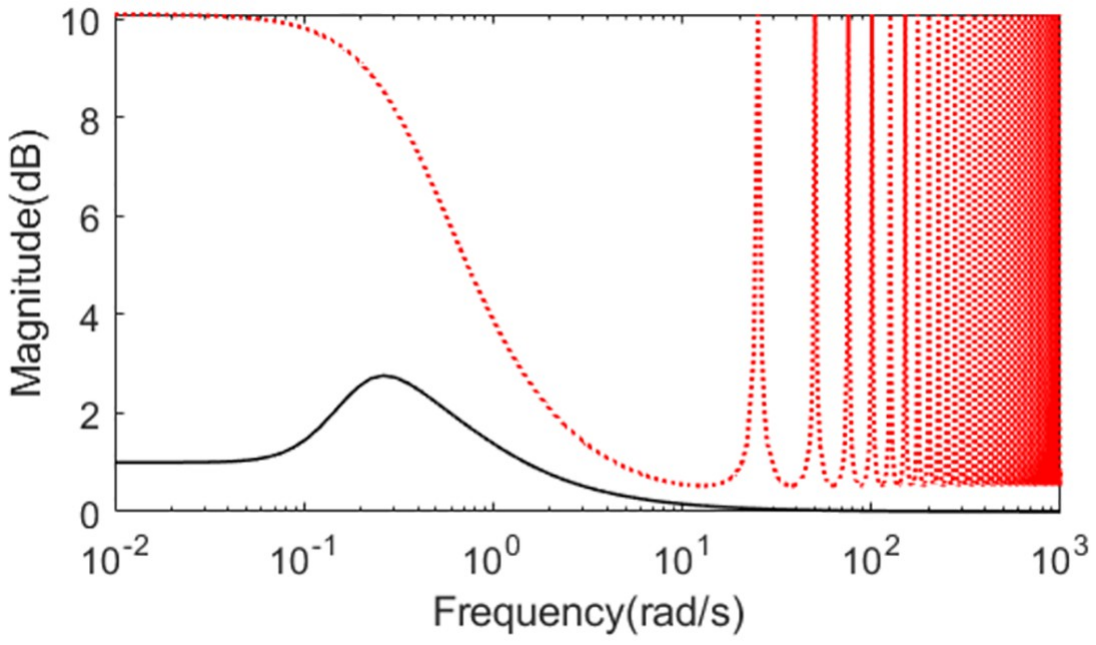
Magnitude of *T*(*s*) of Exa.2.

Uncertainties are introduced to analyze robust performance of the two methods under model mismatching condition. Supposing the actual plant is *P*(*s*) = *e*^−5.25*s*^/(1.1*s*^2^). The responses of the two controllers are illustrated in Figs 11 and 12. It is obvious that the robustness of the presented approach is strong compared with Somak’s method, which is affirmed from the lower values of IAE, ISE, TV and ITAE in Table 2. To demonstrate the robustness, ${\left\|T(s)\right\|}_{\infty }<\left|1/(0.901e^{-0.25s}-1)\right|$ can be got by substituting the assumed perturbations in Eq (28). Fig 13 exhibits the magnitude of *T*(*s*) and the bound causing by the time constant uncertainty Δ_*T*_(*s*). We can observe that the presented control scheme satisfies the criterion of robust stability.

**Table 2:** Evaluation indexes of Exa.2.

State	Method	IAE	ISE	ITAE	TV
Normal	Somak	83.87	228.17	9500	481
	Proposed	69.80	160.67	7770	6
Perturbed	Somak	95.54	256.22	10600	496
	Proposed	75.26	183.78	8090	8.5

**Fig 14:**
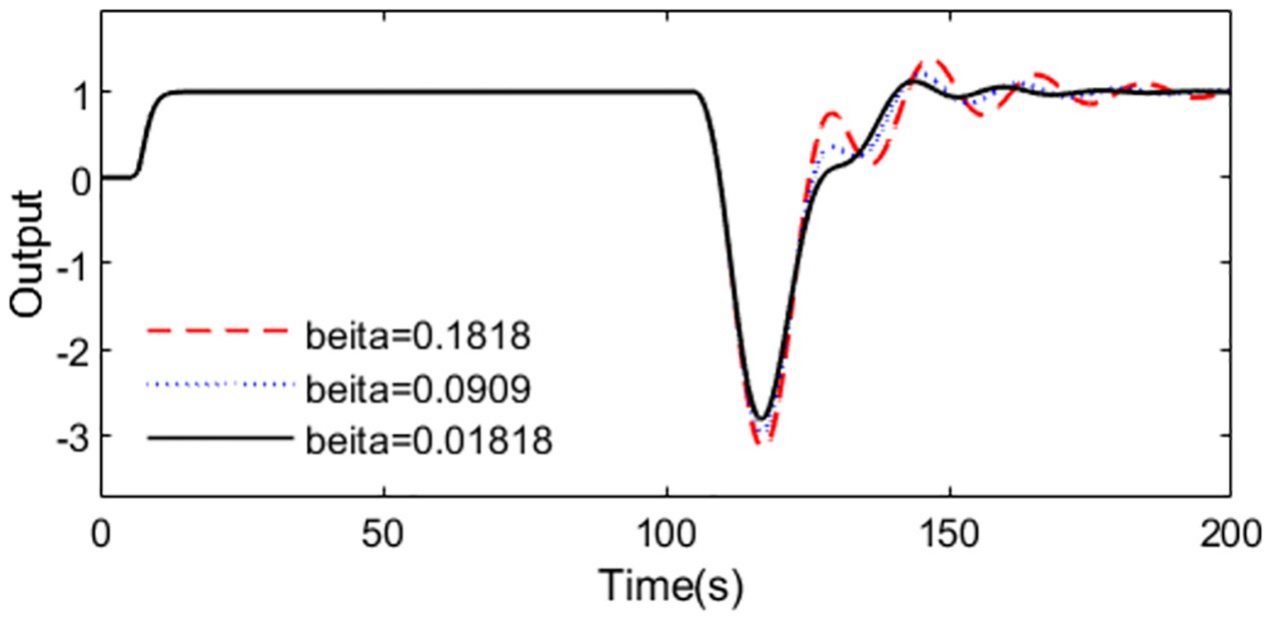
Influence of *ψ* on the performance.

To show the influence of the filter parameter *ψ* on the performance of the normal system, *ψ*, 0.5*ψ*, 0.1*ψ* are considered for the simulation. Supposing with the same other parameters except *ψ*, the system responses to different *ψ* values are shown in Fig 14. We can conclude that little *ψ* will give better disturbance rejection performance but will weaken the robustness of the system.

### Exa.3 First order stable with integrator and time delay

A jacketed continuous stirred tank reactor model is studied here. With the feed concentration *C*_*AF*_ as control input and the reactor temperature *T* as the control output, the detailed system description can be referenced in [4]. On simplifying, the reactor model as follow is adopted

$$P_{m}(s)=\frac{T(s)}{C_{AF}(s)}=\frac{0.9693e^{-s}}{s(12.4224s+1)}$$

To design the servo controller, we set *λ*_1_ = 1 and get *C*_1_(*s*) = 1.064*s*(12.4224*s* + 1)/(*s* + 1)^3^. The process model is approximated as *P*_*m*_(*s*) = −96.93*e*^−*s*^/((−1 + 100*s*)(−1 − 12.4224*s*)), and the disturbance rejection controller can be obtained by selecting *λ*_2_ = 1.5.


$$C_{2}(s)=3.073(1+\frac{0.1424}{s}+2.5658s)\frac{1+0.5s}{1+0.3938s}$$

At the same time, methods proposed by Santosh Kumar [4] and Munna [32] are considered to comparation. Santosh Kumar [4] designed the controller and set point filter as

$$G_{c}=2.7(0.5s+1)(1+3.195s+(1/(16.72s)))/(1+0.2087s),\;F(s)=(1+0.17s)/(1+4.4s)$$

**Fig 15:**
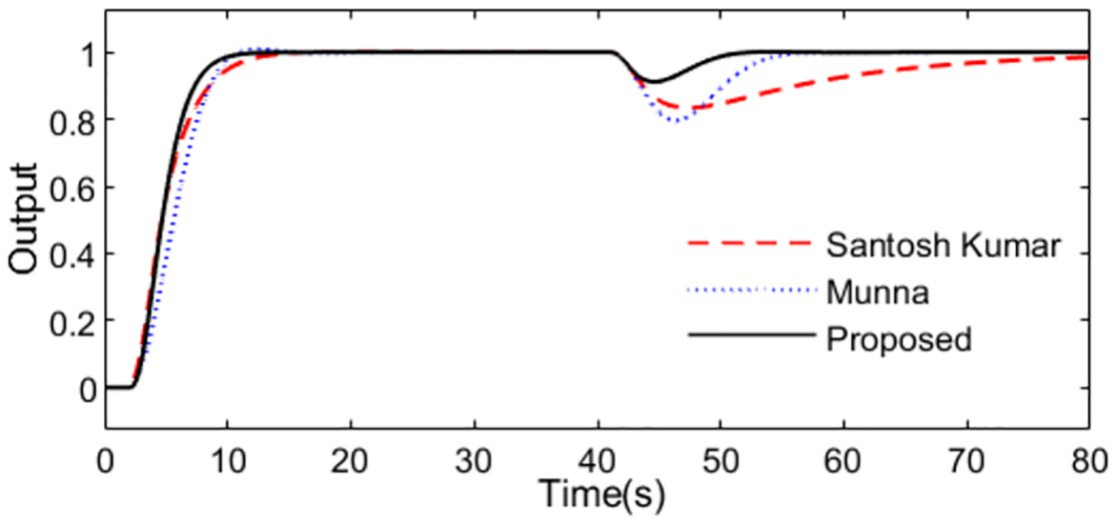
System output in normal case of Exa.3.

**Fig 16:**
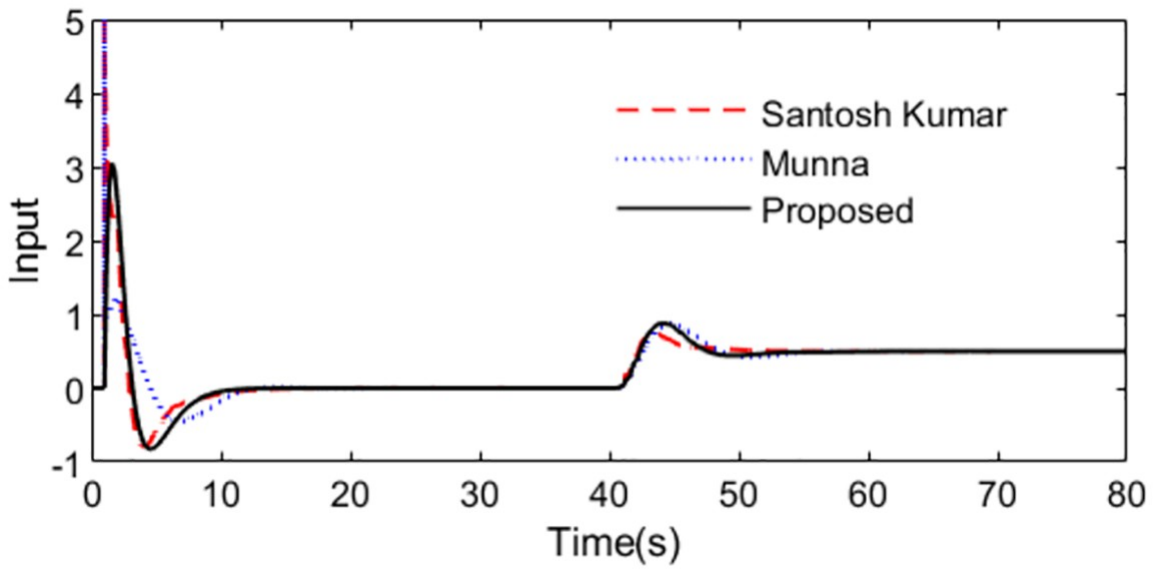
Control action in normal case of Exa.3.

In the control scheme [32], a PID controller *C*(*s*) = 2.594(1 + 1/7.348*s* + 2.7014*s*) and a set point weighting *ε* = 0.35 were designed. With these controller settings, simulations are performed by setting *r*(*s*) as a unit step signal at time t = 1s and setting *d*_*i*_(*s*) as a step signal with magnitude -0.1 at time t = 40s. For perfect model, Fig 15 shows the system outputs, and Fig 16 shows the outputs of the controllers. We can observe that the proposed method exhibits excellent tracking performance and smooth control action.

**Fig 17:**
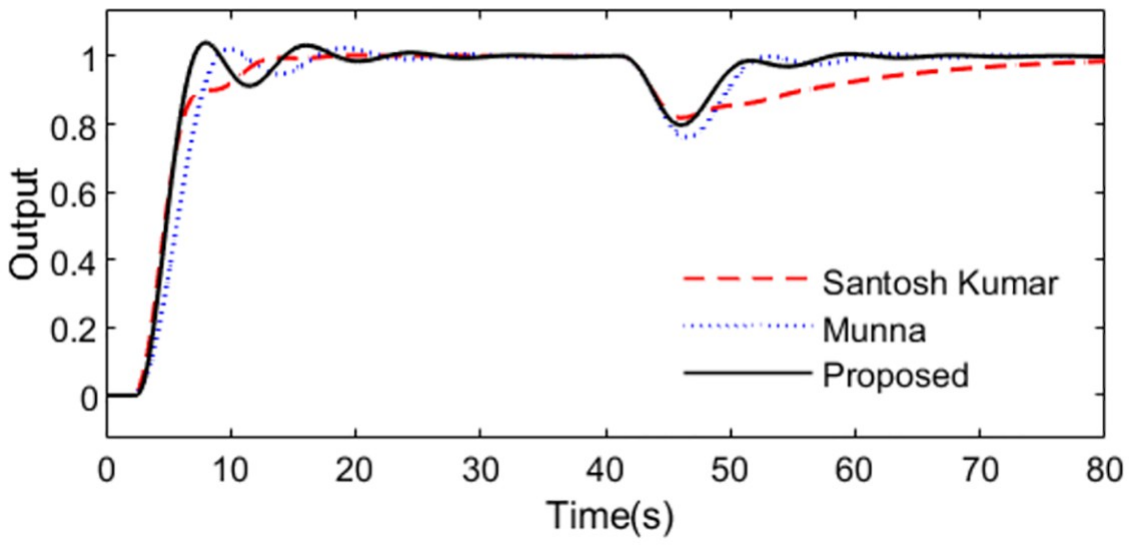
System output in perturbed case of Exa.3.

**Fig 18:**
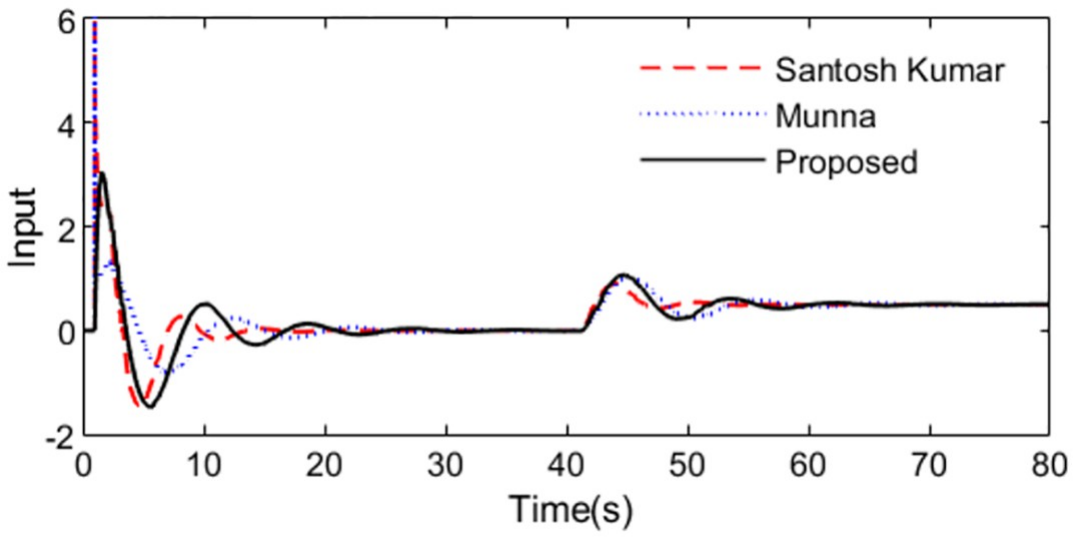
Control action in perturbed case of Exa.3.

**Fig 19:**
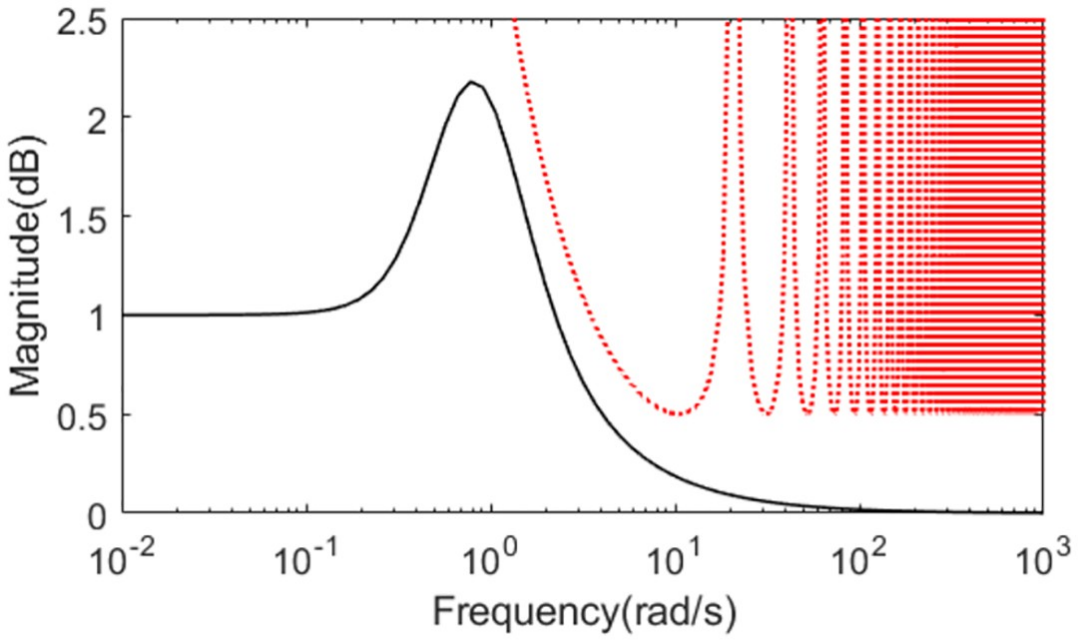
Magnitude of *T*(*s*) of Exa.3.

Further, an error of +30% is assumed in dead time to demonstrate the robust performance for the three methods. Firstly, system outputs and controller outputs in the perturbed condition are demonstrated in Figs 17 and 18. And all the performance indices are calculated in Table 3 for all the methods in normal and perturbed cases. The values of IAE, ITAE, ISE and TV yielded by the presented scheme are relatively small than that by the other two methods. To demonstrate the robustness, we obtain ${\left\|T(s)\right\|}_{\infty }<\left|1/(e^{-0.3s}-1)\right|$ using Eq (27) and the dead time uncertainty. Fig 19 exhibits the magnitude of *T*(*s*) and the bound causing by the dead time uncertainty Δ_*T*_(*s*), from which the conclusion that the presented control scheme satisfies the criterion of robust stability can be drawn.

**Table 3:** Evaluation indexes of Exa.3.

State	Method	IAE	ISE	ITAE	TV
Normal	Santosh	7.22	3.34	176.66	240
	Munna	6.24	3.88	86.02	211
	Proposed	5.15	3.20	67.78	11
Perturbed	Santosh	7.21	3.47	176.38	245
	Munna	6.52	4.05	94.15	220
	Proposed	5.55	3.41	76.47	26

### Exa.4 First order unstable with integrator and time delay

The process with integrator and dead time *P*_*m*_(*s*) = *e*^−0.2*s*^/*s*(*s* − 1) investigated by Ashraf [33] and Santosh Kumar [4] will be studied. For this process, Santosh Kumar designed the controller and set point filter are

$$G_{c}=1.9421(1+1/2.9s+1.5459s)(0.1s+1)/(0.0488s+1)\;\;F(s)=(0.81s^{2}+1.8s+1)/(4.4832s^{2}+2.9s+1)$$

In literature [33], they designed a PID controller *C*(*s*) = 0.87 + 01.19/*s* + 2.42*s* and a filter *F*(*s*) = (2.1*s*^2^ + 2.9*s* + 1)/(12.46*s*^2^ + 4.5*s* + 1). They showed their superiority over many recently reported approaches by simulation. In our presented control scheme, servo controller is obtained as *C*_1_(*s*) = *s*(*s* − 1)/(03*s* + 1)^3^ by setting *λ*_1_ = 0.3. The disturbance rejection controller is got as $C_{2}(s)=2.0546(1+\frac{0.4568}{s}+1.3675s)\frac{1+0.1s}{1+0.01086s}$ by approximating the model as *P*_*m*_(*s*) = 100*e*^−0.2*s*^/(100*s* − 1)(*s* − 1) and by selecting *λ*_2_ = 0.5.

**Fig 20:**
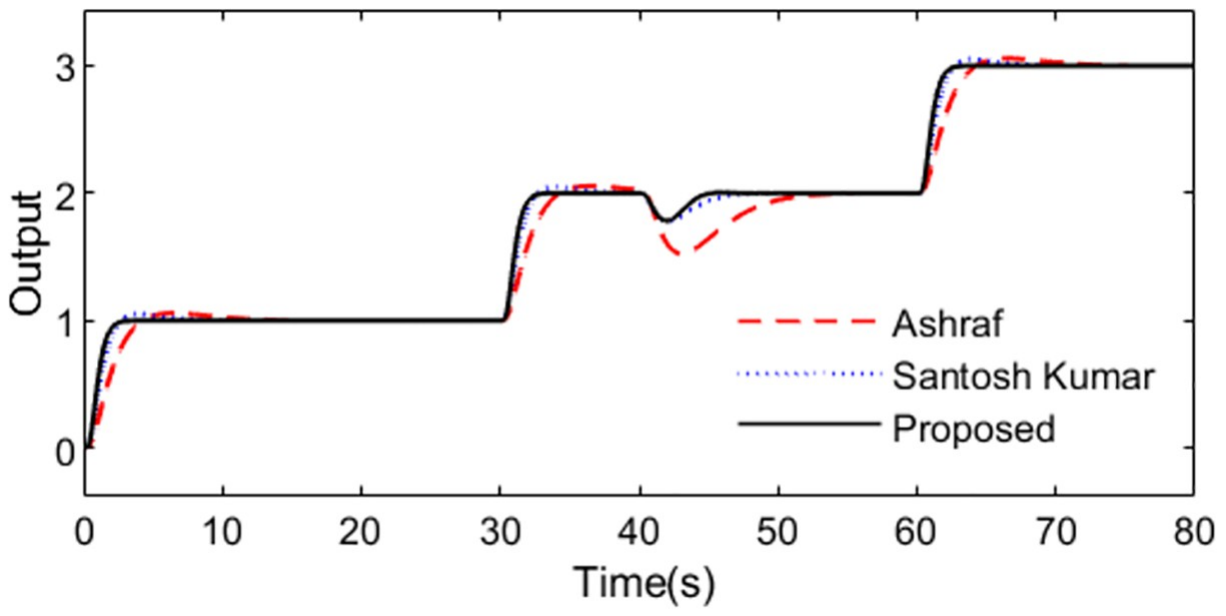
System output in normal case of Exa.4.

**Fig 21:**
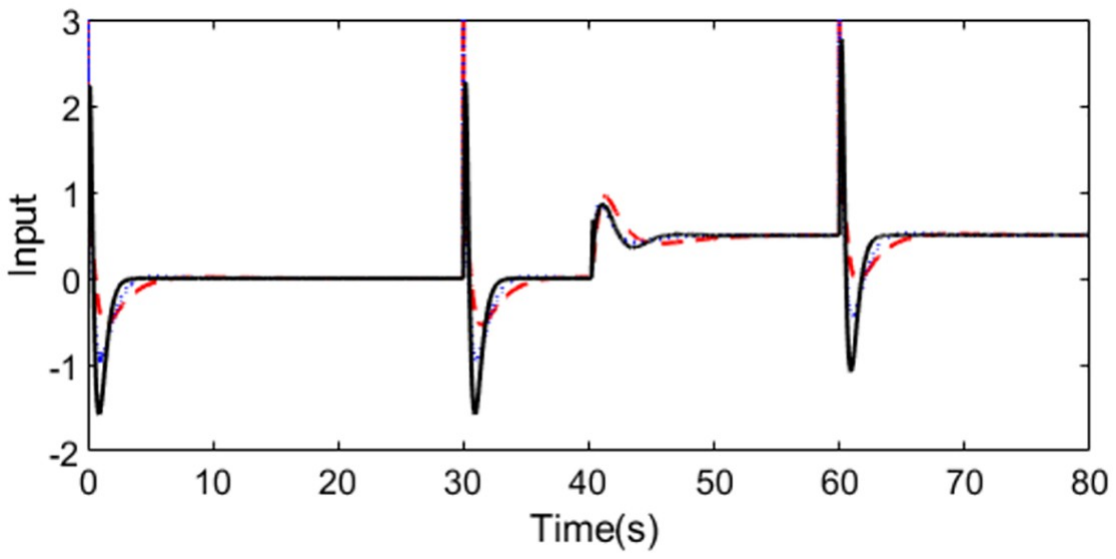
Control action in normal case of Exa.4.

With these controller settings, simulations are performed by setting *r*(*s*) as a staircase signal with period 10s at time t = 1s and setting *d*_*i*_(*s*) as a step signal with magnitude -0.5 at time t = 40s. System outputs and controller outputs are demonstrated in Figs 20 and 21 in the normal case. From the simulation results, it can be easily concluded that the capacity of the presented approach is superior not only in servo response but also in disturbance rejection.

**Fig 22:**
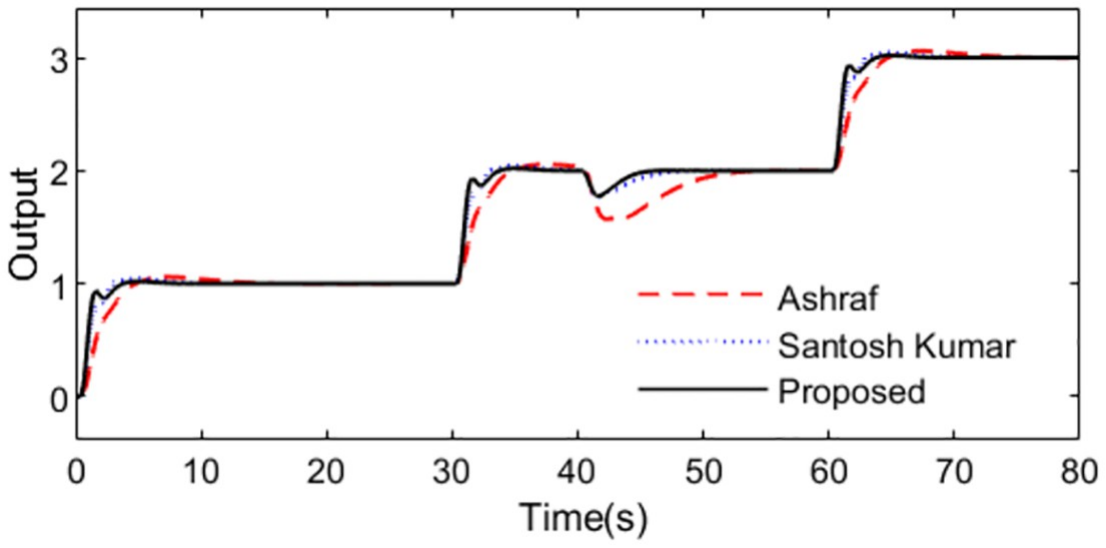
System output in perturbed case of Exa.4.

**Fig 23:**
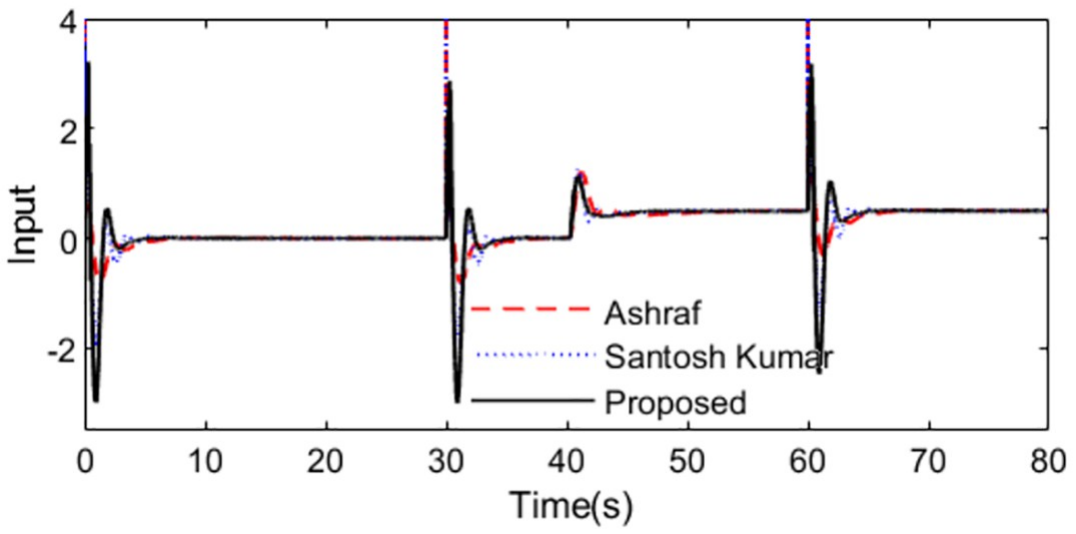
Control action in perturbed case of Exa.4.

**Fig 24:**
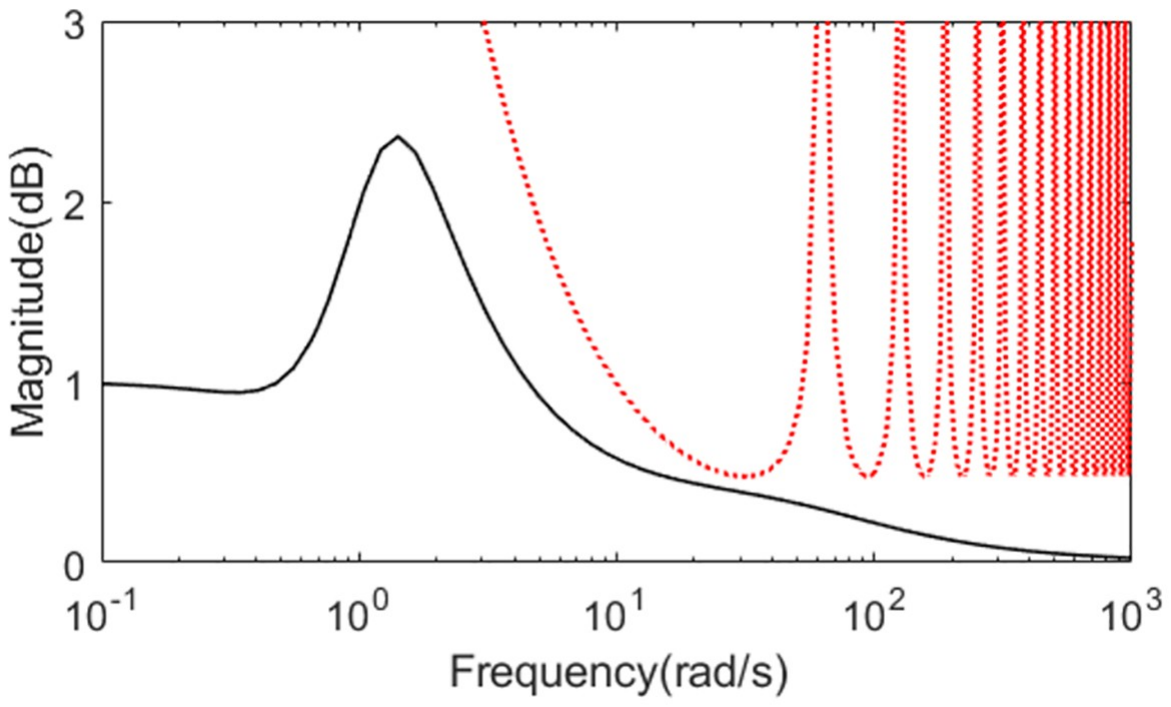
Magnitude of *T*(*s*) of Exa.4.

The perturbed system responses with uncertainties of dead time increasing 50% and gain increasing 10% are given in Figs 22 and 23. Table 4 provides the calculated evaluation index of the performance for all the methods in normal and perturbed cases. From Fig 20 to Fig 23, we can see that the presented control scheme provides satisfied capability in comparison with the other two methods. What’s more, values of IAE, ISE, ITAE and TV in Table 4 are relatively smaller for the presented control scheme in perturbed case as well as in nominal case, which also confirm that the proposed method brings superior control effect. Similarly, we can denote the corresponding uncertainty bound in this case as ${\left\|T(s)\right\|}_{\infty }<\left|1/(1.1e^{-0.1s}-1)\right|$. Fig 24 exhibits the magnitude of *T*(*s*) as well as the uncertainty bound for the proposed method.

**Table 4:** Evaluation indexes of Exa.4.

State	Method	IAE	ISE	ITAE	TV
Normal	Ashraf	9.12	4.94	325.21	579
	Sotosh	4.95	2.87	163.40	1310
	Proposed	3.84	2.54	124.27	43
Perturbed	Ashraf	9.34	4.86	333.92	596
	Sotosh	5.04	2.86	166.67	1521
	Proposed	4.15	2.60	135	266

From the simulation results demonstrated by the four types integrating process, we can see that the performances of set point tracking, disturbance rejection and robustness of the proposed control scheme are excellent. In addition to simpleness, the design method is effective, which is proved by the set point tracking precision from the system response diagram and performance index. Although the plant model is converted to the unstable model in the design procedure for controller *C*_2_(*s*), the performances of disturbance rejection and robustness are still superior.

## Conclusions

A unified control scheme was presented for certain kinds of processes with integrator and dead time including IPTD, DIPTD, FOSITD, FOUITD plants. A simple control structure was designed and desired transfer function was designed for getting the servo controller. IMC theory based analytical controller formulae was employed to obtain the disturbance rejection controller. The system structure and design procedure for the two controllers are simple, and the servo response and disturbance rejection response can be adjusted easily by two independent parameters. Simulations comparing with some outstanding methods were performed to exhibit the capability of the presented control scheme, and some standard plant models were introduced for designing the controllers. Excellent performances of servo, disturbance rejection and robustness in normal and perturbed cases were all demonstrated by the simulation results. Quantitative performance indices including IAE, ISE, ITAE and TV confirmed the advantage of the suggested design approach.
